# Comprehensive two-dimensional ion chromatography (2D-IC) coupled to a post-column photochemical fluorescence detection system for determination of neonicotinoids (imidacloprid and clothianidin) in food samples†

**DOI:** 10.1039/c7ra12555k

**Published:** 2018-03-02

**Authors:** Nadeem Muhammad, Fenglian Wang, Qamar Subhani, Qiming Zhao, Muhammad Abdul Qadir, Hairong Cui, Yan Zhu

**Affiliations:** Department of Environmental Engineering, Wuchang University of Technology Wuhan 430223 China; Department of Chemistry, Xixi Campus, Zhejiang University Hangzhou 310028 China zhuyan@zju.edu.cn +86 571 88823446 +86 571 88273637; Higher Education Department Punjab Lahore Pakistan; Institute of Chemistry, University of the Punjab Lahore Pakistan; College of Pharmacy, Zhejiang Chinese Medical University Hangzhou 310053 China

## Abstract

There are increasing concerns about the dietary risks of neonicotinoids (NNIs); therefore their sensitive and accurate determination in dietary products is indispensable. However, the complex composition of agricultural food matrixes makes their extraction and quantitative determination a challenging task. Realizing this need, we herein report a simple, cost-effective, selective and sensitive fluorescence analytical workflow for analyses of two non-fluorescent neonicotinoids imidacloprid (IMI) and clothianidin (CLT) in six complex food samples (honey, ginger, durian, apple, tomato, cucumber) by online clean-up of sample extracts using two-dimensional ion chromatography (2D-IC) and a subsequent online post column UV induced fluorescence detection system. This online clean-up setup has proven advantageous to improve the limit of detection, potentially diminish matrix effects, and reduce analysis time and labor. The developed method showed excellent analytical figures of merit including linearity, selectivity, repeatability, recovery, and resolution for analysis of IMI and CLT in food samples.

## Abstract 2

A 2D-IC system was successfully fabricated for clean isocratic chromatographic separations and sensitive post column UV induced fluorescence determination of two NNIs in six complex food samples.

## Graphical abstract

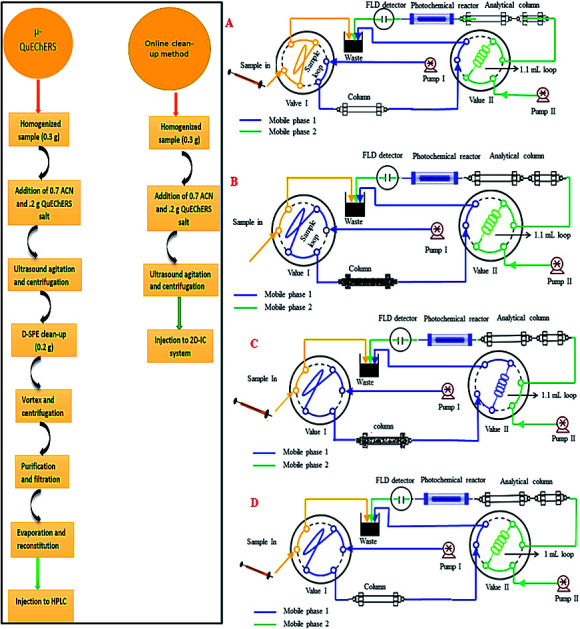

## 1 Introduction

Neonicotinoids (NNI) are structurally similar to nicotine and belong to the class of neurotoxic pesticides. Since the commercialization of imidacloprid in 1985, neonicotinoid insecticides are widely used with an approximate cost of 600 million Euros per year against a variety of biting and sucking insects including whiteflies, beetles, aphids and Lepidoptera species^1–4^ and 60% is used in seed treatments.^5^ The neonicotinoids work as an agonist of the neurotransmitter acetylcholine, which reduces acetylcholinesterase transmission by binding to the post synaptic receptors in the nervous system of living species including insects and humans.^6,7^

On the other hand, there is rising public concern about the dietary risks of NNIs. A number of research studies in the USA and Europe have manifested that even a trace amount of neonicotinoids may cause disorientation, reduce longevity, impair memory and learning, decrease communication and can disrupt the brood cycles of honey bees.^8^ Especially, since last decade there is a dramatic weakness of honeybee hives (*Apis mellifera* L., Hymenoptera: Apidae) and a decrease in production of honey observed due to their contact with the honey bees which lead to their depletion. There is a maximum probability of the existence of their trace residue in honey; and decline of honey production is a threat to global food and medicine due to the growing importance of honey.^9,10^ The European Commission (EU) has banned the use of imidacloprid, clothianidin and thiamethoxam in crops for two years in order to accentuate the awareness of the potentially harmful effect of these pesticides on food products and in honey.^11–13^ Later, to preserve global health and food, the amended European Union legislation has set maximum residue limits (MRLs) for neonicotinoid insecticides in a number of agricultural products between 0.1 and 1 mg kg^−1^ (imidacloprid 0.05 mg kg^−1^ and clothianidin, 0.01 mg kg^−1^, respectively).^2,14–16^

Therefore, to the residual determination of NNIs in agricultural products for better monitoring of food safety, quality and to minimize public health problems; various sensitive, expensive instrumentation being used including gas chromatography coupled with mass spectrometry (GC-MS)^17^ and liquid chromatography braced with tandem mass spectrometry (LC-MS/MS), but due to thermolability, low volatility and high polarity of neonicotinoids residues later one is preferable technique.

However, NNIs polar nature, cost and matrix effect in complex samples make their access and use limited.^18–21^ Whereas, conventional analytical approaches, liquid chromatography coupled with a diode array detector (LC-DAD) and electrochemical detector are cost-effective and simple techniques but poor selectivity and sensitivity have discouraged their application for trace determination of NNIs in complex matrices.^2,17,22–29^ Recently, fluorescence spectrometry appeared as one of the simplest, cost-effective, rapid, sensitive, selective and reliable detection technique, but its application is limited by intrinsic non-fluorescent property of NNIs.^30,31^ This advantageous detection technique was reinstated by direct online photochemical conversion of non-fluorescent analytes into fluorescent species.^32^ The inline photo conversion of analytes is advantageous as it allows exploitation of incomplete reaction (photogenerated unstable radical), improve the efficiency of the photochemical process, minimum use of solvents, minimize generation of waste effluents and save derivatization labor and valuable time.^33^ However, neonicotinoid pesticides only exhibit photochemical fluorescence signal in basic pH, therefore, rare reverse phase chromatographic methods have been reported for determination of IMI only by post-column addition of 0.1 M NaOH.^32^ This extra post-column addition of pump not only make system complex but also increases the chances of baseline drifting, noise, poor repeatability and LOD. This problem to some extent our lab has overcome by introducing direct ion chromatographic based post-column photochemical induced fluorescence determination of pesticides in basic media.^34,35^ Despite all of it, HPLC/MS-MS is advantageous for meanwhile identification of target analyte and the qualifier/quantifier ions. Whereas, in the case of photoinduced fluorimetric determination method unable to identify the possible changes in the structure of target non-fluorescent analytes.

However, this and most of the reported chromatographic methods have been developed for the determination of individual IMD in simple samples (water and soil).^5,23,30^ Therefore, an effective sample preparation is vital not only for analytes pre-concentration but also to remove or minimize other compounds, impurities and matrix interferences without losing analytes of interest. Many off-line sample pretreatment methods have been used to overcome these problems to some extent including solid phase extraction (SPE),^17,25,36^ dispersive solid-phase extraction, dispersive liquid–liquid microextraction,^17,26^ diatomaceous earth-assisted extraction^37^ and ionic liquid phase microextraction.^2^ Most of the above stated sample preparation methods have some major limitations; huge consumption of chemicals and solvent, time-consuming, multiple operation steps required which make these expensive and laborious; whereas the inability to remove matrix interferences of complex samples is a serious limitation. Recently, to compensate the first problem to some extent, there is increasing trend of the development of miniaturized, more efficient, economical and green extraction methods that could significantly reduce the toxic organic solvents and chemical consumption.^38–40^ Particularly, QuEChERS sample preparation method got attentions for extraction of pesticides from complex food and other matrices because of its simplicity, miniaturization, inexpensiveness, efficiency, ruggedness and amenability to high throughput. However, it consists of multiple extraction and cleaning steps; despite that, it is unable to complete elimination of matrix interferences and risk of analyte contamination and loss are maximum during the multiple extraction, evaporation and reconstitution steps; especially, from complex food samples like honey, fruit and vegetables. The natural honey is a sticky and viscous solution having a complex composition with contents of carbohydrates, protein, ash, amino acids, vitamins, enzymes as well as phenolic antioxidants.^10^ Similarly, agricultural products (fruits and vegetables) have high molecular weight and complex interferences like lipids (waxes, triacylglycerols and phospholipids *etc.*), various pigments (carotenoids, chlorophylls, melanoidin *etc.*) and plant resins, which have adversely affected the analysis result by peak masking, false identification of impurity as the analyte peak, impaired detectability, deposition of salt on GC inlet, elution of volatile impurity along with analyte and signal enhancement and suppression. Besides these, QuEChERS method every time needs to modify according to the nature of matrix and analytes of interest.^19,38,39,41–44^ The matrix interferences and analyte loss problems can be resolved by making sample preparation method online with the 2D-IC system just like SPE methods after making online proved more efficient, versatile, reproducible, selective, reduce labor, human error, costs, solvent and time.^45–48^

Since last two decades, an online column-switching technique for heart-cutting is extensively utilized for complex samples clean-up and concentration to reduce matrix interferences.^49–52^ However, most of the reported online extraction techniques use the mini hydrophobic column as pre-treatment column which unable to complete elimination of matrix interferences from analytes of interest and matrices co-elute and transfer along the analytes of interest in second dimension column. This drawback is severe in the case of polar analytes.^32,53^ Whereas, full C18 column as a pretreatment column most of the time help to clean and transfer only single peak to the second dimension.^50^ Therefore, it is need of time to develop an efficient, cheap, selective and sensitive online analytical approach for the determination of more than one neonicotinoids at trace level in complex food samples to access pesticides exposure to human health.^54^ Recently, ion chromatography appeared an efficient and rapid alternative technique for separation and determination of polar analytes apart from ions.^31,43,55^ The electrostatic forces of attraction between polar NNIs and ion exchange columns can be exploited for matric inferences elimination in one dimension and separation of two similar non-fluorescent NNIs in second dimensions with minimum consumption of solvent and time. As far as we know, there are no published reports regarding direct simultaneous separation and determination of two non-fluorescent NNIs (IMI and CLT) by online post column photoinduced fluorescence detection in basic media in complex food samples.

Hence, in order to minimize analyte loss, multiple labor steps, matrix interferences, and to avoid the need of sorbent modification, hereby we report for the first time a systematic online clean-up setup by using 2D-IC column switching technique for the residue analysis of two neonicotinoid pesticides in six complex food samples followed by their sensitive and selective fluorescence determination by exploiting simple post column UV irradiation.

## 2 Experimental

### 2.1 Chemicals

**Fig. 1:**
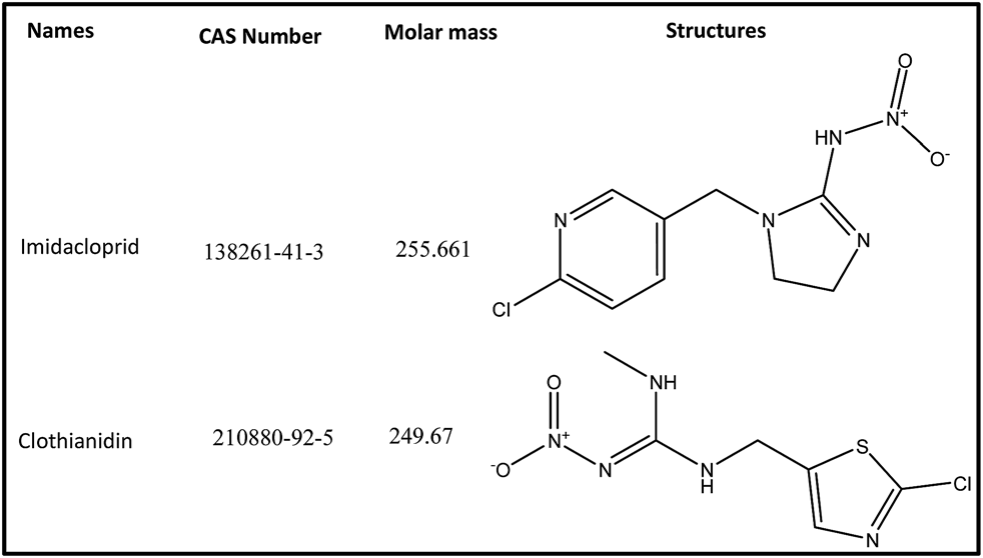
Chemical structures of imidacloprid and clothianidin.

The neonicotinoids imidacloprid (IMD) and clothianidin (CLT) of analytical grade were supplied by Aladdin Chemical Co. Ltd. (Shanghai, China). Their structures and CAS number are given in Fig. 1. The HPLC grade acetonitrile (ACN) and all other chemicals were obtained from Huipu Co. (Hangzhou, China) whereas analytical grade reagents, sodium hydroxide and sodium carbonate for eluent preparation were provided by Thermo-Fisher Scientific (Waltham, MA, USA). The water for experiments and mobile phase preparation was purified by a water purification system (Thermo Fisher Scientific) to a specific resistivity of 18.2 MΩ cm.

### 2.2 Apparatus

The IC-FLD setup consisted of a Thermo Fisher Scientific (Waltham, MA, USA) ICS-1500, which was equipped with a dual-piston serial pump, a Rheodyne (Cotati, CA, USA) Model 08030677 six-port valve adjusted with a 25 μL sampling loop and column heater. An extra 6-port valve switcher was connected with ICS-1500 with a loop of 1.1 mL. The eluent flow rate was 1.0 mL min^−1^. The system was further braced with a model Ultimate 3000 RS fluorescence detector (Dionex, Sunny vale, CA, USA). An extra gradient pump P-680 (Dionex) was used for matrix elimination through pre-treatment column. Two columns IonPac® AS12A column (250 mm × 4 mm i.d; 13 mm particle size) preceded by an IonPac® AG12A guard column (50 mm × 4 mm i.d; 13 mm particle size) and IonPac® AS11-HC column (250 mm × 4 mm i.d; 13 mm particle size) were used for analytical separation of two NNIs and as pre-treatment column for matrix elimination from complex samples extract, respectively.

A home-made photo chemical reactor was designed by coiling the knitted PTFE tube (17 cm long × 1.5 mm o.d. × 0.5 mm i.d.) around a 20 cm long low-pressure mercury lamp (18 W, 254 nm). It was further fitted into a PVC cylinder and internally covered with aluminum foil to have back the maximum UV light reflection and to minimize light dissipation in surrounding environment. The data analysis was performed with a personal computer equipped with Chromeleon 7.2 software (Thermo-Fisher Scientific, Waltham, MA, USA). A precise pH-meter (Mettler Toledo, FE20 and China) was used for the pH measurements. The SB-5200DT ultrasonic cleaner (Scientz Biotechnology Co. Ltd., Ningbo, China) was used for sonication of sample and eluent. The agricultural samples ginger, durian, apple, tomato and cucumber were bought from a local market in Hangzhou (China) and stored at 4 °C. The third, multifloral pure honey sample was cultivated under our surveillance in Punjab, Pakistan, which was stored in an amber vial at 4 °C in darkness.

### 2.3 Solutions

**Fig. 2:**
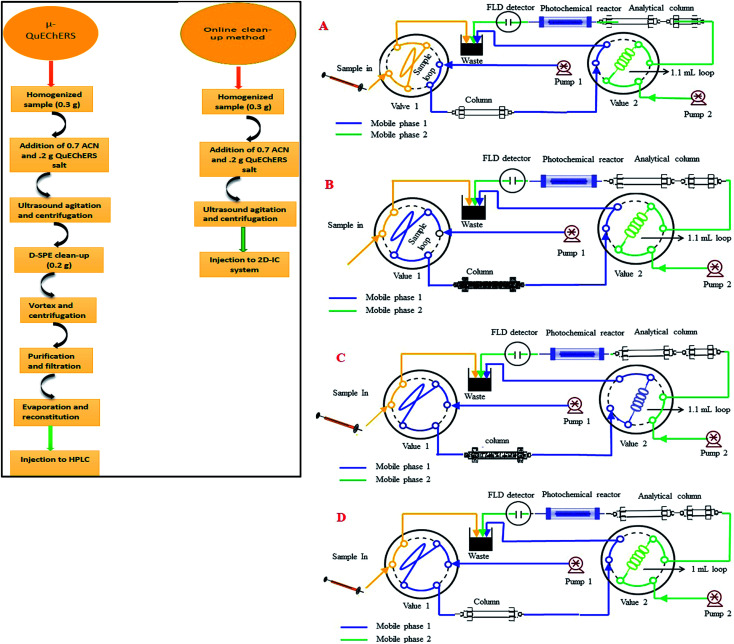
Systematic flow scheme of 2D-IC system: (A) sample loading; (B) matrix interferences removal; (C) analytes transfer in second dimension and (D) regeneration, re-equilibrium of pre-treatment column and isocratic separation and post column PIF detection in second dimension.

The stock solutions of two NNIs (100 mg kg^−1^) were prepared by precise weighing and separately dissolving corresponding amounts in a mixture of water–acetonitrile (50 : 50) in a 100 mL amber glass bottles and stored at 4 °C in darkness. Working standard solutions were prepared every day in mobile phase for method validation. The calibration standard solutions were prepared in the concentration range of 12–1500 μg kg^−1^ of IMI and 84–5000 μg kg^−1^ for CLT at six concentration level. All standard and sample solutions were injected through Millipore membrane PTFE filter (0.45 μm particle size) into the 2D-IC system. Two mobile phase systems were prepared, mobile phase (M.P) B: 1.5 mM Na_2_CO_3_ + 20 mM NaOH + 18% ACN was used for isocratic separation of two NNIs and mobile phase (M.P) A: 50 mM NaOH + 20% ACN was used for online matrix interferences elimination from injected matrix match samples extracts and for regeneration of pre-treatment column as shown in Fig. 2.

### 2.4 Sample preparation

P. Porto-Figueira *et al.*, optimized a μ-QuEChERS method for extraction of pesticides from cereals. It consists of two main extraction and dispersion steps.^39^ It's modified extraction steps were utilized for the extraction of IMI and CLT from six complex samples including honey, ginger, durian, apple, tomato and cucumber. Briefly, it involved precise weighing and homogenizing of QuEChERS mixture consisting of 0.12 g anhydrous magnesium sulfate and 0.07 g sodium chloride. The 0.3 g of each homogenized samples were transferred to 4 mL centrifuge tube with subsequent addition of 0.9 mL of ACN and 0.2 g of QuEChERS mixture. The tube carrying multi composition mixture (sample + salts + solvent) was shaken by hand for 30 s, followed by ultrasound agitation (SB-5200DT ultrasonic cleaner Scientz Biotechnology Co. Ltd., Ningbo, China) for 10 min and finally centrifuged (Hitachi CF15RXII high-speed centrifuge with a T15A42 rotor, Tokyo, Japan) at 6500 rpm for 5 min. The organic layer was decanted into another dry vial for injection into the 2D-IC system for online elimination of matrices interferences and PIF sensitive determination of IMI and CLT in complex sample extracts.

### 2.5 General online operational procedure of 2D-IC system

The online operational procedure of 2D-IC system to eliminate matrix interferences from six complex sample extracts and simultaneous isocratic separation of two NNIs along their post column online photo induce fluorescence (PIF) determination comprised of four steps: (a) sampling; (b) online sample pretreatment; (c) collection of target analytes and (d) analyzing the NNIs and re-equilibrium of the pretreatment column for subsequent analysis.

Firstly, the sample was manually loaded into the 25 μL sample loop of valve 1 (Fig. 2A), and it is carried to the pretreatment column Ionpac AS11-HC by pumping mobile phase A (55 mM NaOH + 20% ACN) by HPLC pump A. Most of the high molecular weight hydrophobic matrixes interferences were quickly discarded into waste, while both polar NNIs were retained into the AS11-HC column and after 4.50 min the value 2 having 1.1 mL loop turn into load position and start collecting target analytes within 1.0 min time span (Fig. 2B and C). After 1.0 min of analyte collection, the NNIs from the collection loop were delivered to the analytical column Ionpac AS12A protected by a guard column Ionpac AG12A with mobile phase B (1.5 mM Na_2_CO_3_ + 15 mM NaOH + 18% ACN) for their isocratic separation (Fig. 2D). Meanwhile, the pretreatment column was washed and regenerated by high strength mobile A. After 9.0 min the valve 1 switch back to load position and valve 2 switches to inject position to bring the 2D-IC system come back to the initial state (Fig. 2A) and to achieve the equilibrium for the subsequent sample analysis. The complete analytical procedure comprised of 31 min, in which 15.5 min for offline solid–liquid extraction of two analytes from complex samples and 15.5 min for their online clean-up and separation followed by photoinduced fluorescence detection. The complete operation procedure of 2D-IC setup is given in Table 1.

**Table 1:** Automatic system operation procedure^b^

Position	Programming	Time (min)	Mobile phase of pre-treatment column	Mobile phase of analytical column	Valve 1 (V-1)	Valve 2 (V-2)
1	Sampling^a^	0.0	55 mM NaOH + 20% ACN	1.5 mM Na_2_CO_3_ +15 mM NaOH + 18% ACN	Load	Inject
2	Sample extract pre-treatment	0–4.50	—	—	Inject	Inject
3	Analytes collection	4.50–5.50	—	—	Load	Load
4	Analyses, regeneration, and re-equilibrium	5.50–15.50	—	—	Load	Inject

a Manual 25 μL sample injection.

b Inject for 60 s.

### 2.6 Analytical figures of merit

Several agencies have suggested guidelines to validate a new analytical method. By following the SANTE/11945/2015 guidelines, a few key parameters; linearity, selectivity, sensitivity, within-run and between-run precision, accuracy, ruggedness, matrix effect, limit of detection (LOD) and limit of quantification (LOQ) were properly validated.

The linearity of the detector response for both NNIs was assessed under the optimal conditions from the standard and matrices matched calibrations standard at six concentration level in the range 12–5000 μg kg^−1^ for IMI and CLT. The selectivity of this method was observed by duplicate injection of blank samples extracts to observe the influence of matrix effect at a retention time of both analytes.^56^ The method limit of detections and quantification were estimated as the lowest concentration resulting in a signal-to-noise ratio equal to 3 and 10-fold, respectively. The LOQs were used as the lowest concentration at the calibration curve according to SANTE/11945/2015 guidelines.^57^ The precision of the method was determined at matrices matched three concentration levels LOQ, 2× LOQ and 10× LOQ and their relative standard deviations (%RSD) were computed as a measurement of the precision. In addition, due to the complexity of the matrices the intra- and inter-day precisions were also calculated in quintet within one day and over three days, respectively. The accuracy of the method was calculated as mean recoveries by spiking μ-QuEChERS extract at three level (LOQ, 2× LOQ and 10× LOQ) in the quintet.

All recoveries (%) were calculated by following equation.Recovery (%) = (*C*_1_ – *C*_2_)/*C*_3_ × 100.*C*_1_ = concentration in a matrix extract spiked prior to μ-QuEChERS extraction method, *C*_2_ = concentration in unfortified matrix extract obtained by QuEChERS extraction method, *C*_3_ = concentration of fortification (standard solution).

Matrix effect (ME) was determined by comparing the slope ratios of the two sets; matrix matched (honey, durian, ginger, apple, tomato and cucumber) and pure solvent calibration curve obtained at six level of concentration for IMI and CLO. According to guidelines; matrix effect value is within the range of ±20, the results are acceptable.ME (%) = (slope of calibration curve in matrix/slope of calibration curve in solvent − 1) × 100.

## 3 Results and discussion

### 3.1 Optimization of photochemical derivatization

The post-column photo derivatization is advantageous due to its simplicity, online derivatization, short time, no need of long-time stability of photo products, original analytes structure and retention time remains same before and after online photochemical derivatization and both analytical selectivity and sensitivity of analytes increases. Both IMI and CLT do not show intrinsic fluorescence in aqueous medium.^32^ The photochemical behavior of each individual NNIs was evaluated using aqueous mobile phase with different solvents (acetonitrile, methanol, ethanol). It observed both analytes were unreactive toward photochemical reaction and did not show any fluorescence signal. The previous studies also revealed that IMI has shown fluorescence signal only after post column addition of 0.1 M NaOH.^32,34^ Therefore, ion chromatographic based post column UV induce fluorescence determination was carried out for the determination of two non-fluorescent neonicotinoids.

There are various factors including nature of the basic medium, pH, solvent, and irradiation time were optimized in order to establish the optimum condition for the sensitive determination of NNIs by exploiting post column photo induced fluorescence determination in complex samples. All studies were performed by varying each respective variable in return keeping the others constant. Firstly, it is important to know optimum excitation and emission wavelength in order to have their maximum fluorescence intensity. Both analytes turn by turn scanned by passing online through the homemade photochemical reactor. It was observed IMI and CLT showed big peaks at *λ*_ex_/*λ*_em_ = 332/368 nm and *λ*_ex_/*λ*_em_ = 353/401 nm, respectively as shown in Fig. S1(a).†

A flow injection analysis was conducted to investigate the effect of basic media on the photoinduced fluoresce (PIF) intensity of NNIs. To observe this phenomenon various bases and their different IC column compatible combinations with each other (NaOH, KOH, Na_2_CO_3_, NaHCO_3_, Na_2_CO_3_ + NaHCO_3_, NaOH + NaHCO_3_ and NaOH + Na_2_CO_3_) were prepared with the constant addition of 18% ACN. It was observed IMI exhibited much high PIF intensity as compare to CLT in all bases, this probably due to the presence of the aromatic ring in IMI structure. The ionization of alpha proton in basic media extends the resonance path up to two rings which helped in to have their high PIF intensity. Both NNIs showed high PIF intensity in the strongly basic mobile phase of KOH and NaOH as compared to weakly basic Na_2_CO_3_ and NaHCO_3_. Whereas, CLT showed significantly higher PIF signal in the weakly basic medium in contrary to IMI. However, both CLT and IMI simultaneously showed high fluorescence intensity in a combination of NaOH and Na_2_CO_3_ or NaOH and NaHCO_3_ as depicts in Fig. S1(b).† Especially, CLT PIF signal increased two times in medium composed of strong and weak base (NaOH, Na_2_CO_3_) as compared to only strong base (NaOH, KOH). Therefore, weak and strong base combination (NaOH + Na_2_CO_3_) was used as a mobile phase for the separation and analysis of both NNIs in order to have their optimum PIF intensity.

The influence of pH on both NNIs PIF intensity was investigated by changing the composition of mobile phase with NaOH and Na_2_CO_3_ to have pH in the range of 7–14. A significant increase in PIF intensity of both NNIs was observed as pH increased from 6 to 14 and became maximum at pH 11–12. This effect was more dramatic in the case of IMI, while CLT PIF intensity slowly increased and became constant as shown in Fig. S2(a).† Therefore, the pH 11–12 was selected as optimum pH for their sensitive determination. This effect probably due to ease of NNIs degradation in basic media under UV light and changed into fluorescent species.

The PTFE capillaries have been used for the UV induce fluorescence. Therefore, the residence time of analytes of interest under UV light is very critical to have their optimum fluorescence intensity. The residence time of NNIs was controlled by variation of PTFE coil length and it observed IMI PIF intensity increases as its residence time elongated from 18 to 30 s after that it slowly decreased and became constant, while CLT displayed maximum PIF intensity for 18 s and then it remains almost constant as shown in Fig. S2(b)†.

### 3.2 Switching parameters: IC clean up and separation

#### 3.2.1 Chromatographic separation conditions

The NNIs polar nature and their property to show PIF intensity in basic medium motivated us to investigate their separation through anion exchange columns. During the experimental work, multiple anion exchange column including IonPacs AS11-HC, IonPacs As16A, IonPacs AS14A and IonPacs AS12A were tested for their isocratic separation. The first three column behave similarly, all face the problem of poor peak shape of IMI and unsatisfactory peak resolution. To improve the peak shape of IMI the concentration of NaOH and ACN were increased, however soon after IMI and CLT peak start overlapping at high strength mobile phase, whereas at weak strength mobile phase IMI shown broad peak width and suffer from peak tailing. Both polar nature and a hydrophobic aromatic moiety of NNIs exploited multiple interactions including π–π interactions, dipole–dipole interactions, hydrophobic and electrostatic interactions with the anion exchange stationary phase and its PS/DVB copolymers. These interactions simultaneously operate and help to resolve and isolate both analytes isocratically.

**Fig. 3:**
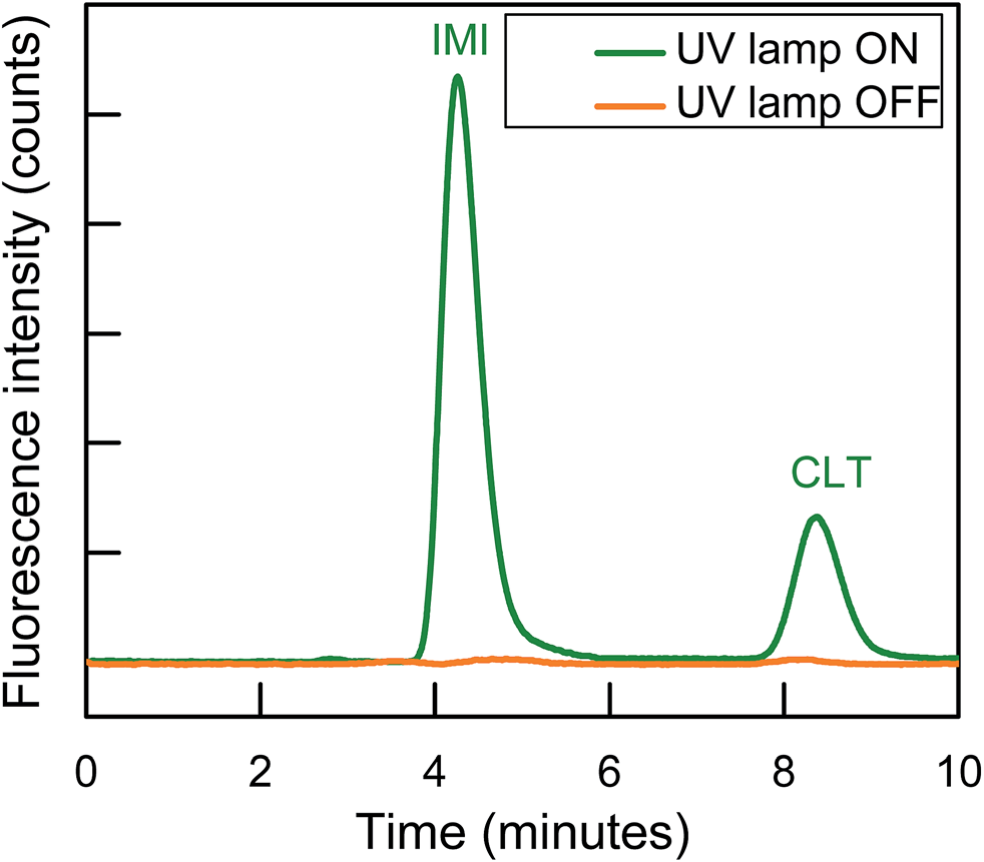
Isocratic separation of two NNIs and PIF detection at *λ*_ex_/*λ*_em_ = 332/368 nm for IMI and *λ*_ex_/*λ*_em_ = 353/408 nm for CLT, respectively.

Finally, IonPacs AS12A was successfully utilized for their isocratic separation by using only 18% ACN to improve peak shape and to finish separation within 10 min. Therefore, 1.5 mM Na_2_CO_3_ + 15 mM NaOH + 18% ACN was used as a mobile phase to gradual overcome all type of these interactions and to separate them isocratically as shown in Fig. 3.

### 3.3 Optimization of the online 2D-IC operation procedure

The simplification and online sample clean-up with 2D-IC fluorimetric analysis would be advantageous from an analytical perspective as cost-effective, time-saving, less laborious, enhance analytical column life and environmental friendly for the complex food samples analysis. The food samples have a complex composition as detailed in the Introduction, their direct injection not only can cause matrix effect for accurate determination of an analyte of interest but also these might can damage the analytical column within days. Therefore, a 2D-IC system was fabricated to overcome these problems, which consists of a pair of HPLC pump, column, and two valves, in which V-1 is fit together with 25 μL sample loop and another V-2 was assembled with 1.1 mL sample collection loop as shown in Fig. 2.

### 3.4 Online removal of the matrices from samples extracts

**Fig. 4:**
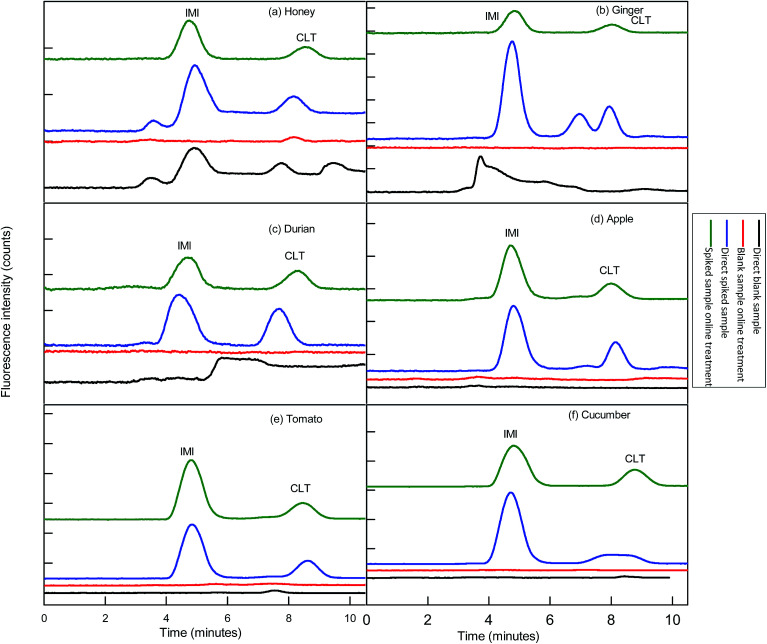
Sample chromatograms: analyses of blank samples extract without 2D-IC treatment (black lines); analyses of fortified sample extract at (10× LOQ) fortification level without 2D-IC treatment (blue lines); analyses of blank sample extract *via* 2D-IC treatment (red lines) and analyses of fortified sample extracts *via* 2D-IC treatment (green lines). Mobile phase: 1.5 mM Na_2_CO_3_ + 15 mM NaOH +18% ACN; columns: IonPac® AS12A column (250 mm × 4 mm i.d; 13 mm particle size) preceded by an IonPac® AG12A guard column (50 mm × 4 mm i.d; 13 mm particle size); injection volume: 25 μL; temperature: ambient; flow rate: 1.0 mL min^−1^; FLD detector wavelengths: *λ*_ex_/*λ*_em_ = 332/368 nm for 0–6.50 min and 353/408 nm for 6.5–10 min; peaks: (1) IMI = imidacloprid and CLT = clothianidin.

Before functionalization of the 2D-IC system for the removal of matrix interferences of three sample extracts, multiple switching time of the system were precisely determined. This could only possible by knowing the elution time of all samples extract matrixes on the pre-treatment column, which were determined by directly connecting the column to the FLD detector. For this purpose mobile phase A composition was optimized by variation of NaOH concentration and ACN content and it was observed high strength mobile phase A (55 mM + 20% ACN) help to discard maximum matrix interferences into waste without losing analyte of interest within 4.5 min, and meanwhile both analytes were also simultaneously eluted in form of one peak which was directly collected into a 1.1 mL loop of V-2 which switches to load position just after 4.50 min as shown in Fig. 2B. Exactly, after 1 min of analyte collection the IC separation started by injected the matrix-free sample for 60 s in second dimension analytical column with the continuous pumping of mobile phase B (1.5 mM Na_2_CO_3_ + 15 mM NaOH + 18% ACN) by HPLC pump 2 as shown in Fig. 2C. Meanwhile, pretreatment column during same IC separation time undergoes through regeneration and re-equilibrium process with mobile phase A and switched to the original position for subsequent sample analysis as the systematic procedure is shown in Table 1 and Fig. 2A–C. The clean separation and analysis of CLT and IMI in three complex samples (honey, ginger, durian, apple, tomato and cucumber) is displayed in Fig. 4.

### 3.5 Analytical figure of merits

The developed 2D-IC method has been validated by following SANTE/11945/2015 guidelines for qualitative identification purpose. The method linearity was estimated over concentrations range 12–5000 μg kg^−1^ according to PIF intensity detection sensitivity of individual NNIs in basic media. The excellent correlation coefficient *r*^2^ ≥ 0.999 of both NNIs are obtained as given in Table 2.

**Table 2:** Calibration and peak parameters for nicotinoids in a solvent

Analyte	Retention time (min)	Peak asymmetry	Peak resolution	Linear range (μg kg^−1^)	Correlation (*r*^2^)
IMI	4.90	1.32	4.29	0.12–1250	0.9999
CLT	8.90	1.31	—	0.51–5000	0.9991

The LODs and LOQs of both NNIs in three samples were retrieved in the range from 0.035–0.154 μg kg^−1^ and 0.120–0.512 μg kg^−1^, respectively as given in Table 3. These low LODs are the result of high PIF intensity and maximum recoveries of analytes of interest due to the online clean-up of matrix interferences from sample extracts by using the 2D-IC system.

**Table 3:** LODs and LOQs for two NNIs in three matrices

Analyte	Honey (μg kg^−1^)		Ginger (μg kg^−1^)		Durian (μg kg^−1^)		Apple (μg kg^−1^)		Tomato (μg kg^−1^)		Cucumber (μg kg^−1^)	
	LOD	LOQ	LOD	LOQ	LOD	LOQ	LOD	LOQ	LOD	LOQ	LOD	LOQ
IMI	0.036	0.119	0.035	0.119	0.037	0.123	0.034	0.113	0.35	0.116	0.037	0.123
CLT	0.153	0.510	0.151	0.502	0.154	0.512	0.142	0.472	0.143	0.476	0.144	0.479

The intra- and inter-day precisions of the method at three levels are expressed as the %RSD of the relative peak areas of the day controlled fortified samples. Both intra- and inter-day precisions values were within the range 0.12–12.69% and 2.16–12.36%, respectively as given in Table 4. The high precision of the method reflects the reproducibility of the method. The accuracy of the method was gauged in fortified blank samples at three concentration levels LOQ, 2× LOQ and 10× LOQ in the quintet and good recoveries were obtained in the range 82.0–106% as given in Table 5.

**Table 4:** Intra- and inter-day precision at three concentration levels LOQ, 2 × LOQ, 10 × LOQ for IMI and CLT in honey, ginger, durian, apple, tomato and cucumber samples. (*n* = 5)

Analyte	Conc. level (μg kg^−1^)	Honey (% RSDs)		Ginger (% RSDs)		Durian (% RSDs)		Apple (% RSDs)		Tomato (% RSDs)		Cucumber (% RSDs)	
		Intra	Inter	Intra	inter	Intra	Inter	Intra	Inter	Intra	Inter	Intra	Inter
IMI	LOQ (0.12)	0.12	2.60	5.18	8.96	1.16	8.68	1.25	3.68	5.6	7.52	0.78	3.07
	2× LOQ (0.24)	10.31	3.69	2.16	4.70	12.69	9.73	5.81	8.54	8.89	7.23	0.21	5.39
	10× LOQ (1.2)	6.14	5.98	7.96	5.23	8.98	8.90	3.69	9.27	0.86	2.37	5.86	2.17
CLT	LOQ (0.51)	7.96	10.23	10.44	12.36	0.12	5.67	6.18	3.84	1.24	4.21	3.57	8.42
	2× LOQ (1.02)	9.38	11.40	4.16	7.23	3.19	7.65	7.23	4.87	5.98	8.71	2.79	8.12
	10× LOQ (5.1)	0.68	9.51	3.24	5.16	5.26	11.89	8.95	4.20	0.95	3.58	0.91	4.81

**Table 5:** Recovery (%RE) and repeatability (%RSD) at three concentration LOQ, 2× LOQ, 10× LOQ for IMI and CLT in honey, ginger, durian, apple, tomato and cucumber samples (*n*^a^ = 5)

Analytes	Con. (μg kg^−1^)	Honey	Ginger	Durian	Apple	Tomato	Cucumber
IMI	LOQ (0.12)	77.41 ± 0.12	97.22 ± 5.18	93.23 ± 1.16	84.14 ± 7.13	101.24 ± 8.74	99.73 ± 3.16
	2× LOQ (0.24)	79.80 ± 10.31	100.46 ± 2.16	105.6 ± 12.69	86.10 ± 9.53	100.13 ± 7.12	101.2 ± 10.79
	10× LOQ (1.2)	89.36 ± 6.14	84.50 ± 7.96	83.18 ± 8.98	82.43 ± 5.24	94.50 ± 7.06	93.12 ± 7.38
CLT	LOQ (0.51)	81.10 ± 7.96	83.18 ± 10.44	90.06 ± 0.12	81.80 ± 5.06	93.12 ± 11.04	98.03 ± 0.18
	2× LOQ (1.02)	95.23 ± 9.38	88.32 ± 4.16	106.8 ± 3.19	89.28 ± 10.18	88.72 ± 7.16	102.8 ± 8.13
	10× LOQ (5.1)	90.75 ± 0.68	74.60 ± 3.24	73.71 ± 5.26	90.05 ± 1.73	84.80 ± 4.94	83.21 ± 3.29

a Replicate of five readings.

One of the most important parameter of method validation and this method development is the matrix effect. It was the fundamental purpose of this method development to minimize the matrix effect in order to avoid the camouflage determination of NNIs in complex samples analysis. The blank samples extracts obtained by μ-QuEChERS method were fortified and matrix effect (ME) were calculated by using the matrix effect equation as detailed in Section 2.6. It was observed matrix effect was comprehensively reduced as all ME values are within the range of ±20% by integration of μ-QuEChERS method to the 2D-IC system as given in Table 6.

**Table 6:** Matrix effects (ME) and determination coefficients (*r*^2^) for NNIs in six matrices^a^

Analyte	Honey		Ginger		Durian		Apple		Tomato		Cucumber	
	ME	*r* ^2^	ME	*r* ^2^	ME	*r* ^2^	ME	*r* ^2^	ME	*r* ^2^	ME	*r* ^2^
IMI	−0.22	0.9993	10.02	1	−14.21	0.9999	3.52	0.9980	5.78	0.9934	−0.30	1
CLT	+11.90	0.9963	+5.30	0.9990	+11.06	1	1.23	0.9974	2.98	0.9945	0.40	0.9999

a IMI: imidacloprid and CLT: clothadine.

This developed method also displayed excellent selectivity after integration of μ-QuEChERS sample preparation method to the 2D-IC system as shown in Fig. 4a–c. The good sensitivity of the method can be observed from the all excellent determination of coefficient (*r*^2^) values ≥0.9999 and the low LOD values of both NNIs as given in Table 2. In addition, clean separation and analyses of both NNIs in complex samples are shown in Fig. 4 and comparison of this developed method with other is also given in Table 7.

**Table 7:** Comparison of sample preparation and detection methods for the determination of imidacloprid and clothianidin in food samples^a^

Sample	Sample preparation method	No. of analytes	Solvent uses (mL)	Analysis time (min)	Detection	LOD	References
Honey	DLLME and QuEChERS	7	>2.5, >10	66	HPLC-UV	1.5–2.5 μg kg^−1^	54
Cucumber	QuEChERS method	4	>10	20	HPLC-DAD	0.04 mg kg^−1^	1
Eggplant, cucumber	Ionic liquid extraction	7	>33	30	HPLC-DAD	0.002–0.003 mg kg^−1^	25
Honey	Ionic liquid extraction	4	>50 μL	14	HPLC-DAD	50 μg kg^−1^	58
Honey	SPE	7	>19	No separation	LC/LC-MS	0.03 μg kg^−1^	59
Potato	SPE	4	>80	9.10	LC-DAD	3.2–15 μg L^−1^	60
Cucumber soil	QuEChERS method	3	>10	14	LC-DAD	0.01–0.08 mg kg^−1^	61
Commercial formulation	Direct	2	N.A	N.A	Spectrophotometric	0.17–0.32 mg L^−1^	62
Honey bee	SPE	2	>85 mL	7	HPLC-FLD	0.09–1.5 μg kg^−1^	32
Honey bee, maize leave	Sample clean-up	2	>50–150 mL	25	LC-hν-ED	2.4 mg L^−1^	53
	SPE	2	100 mL	7	MEKC-UV	0.71–1.18 mg L^−1^	63
Honey, ginger, durian	Online-sample clean-up	2	900 μL	9.5	IC-hv*-*FLD	0.036–0.15 μg kg^−1^	This work

a DLLME: dispersive liquid–liquid microextraction; MEKC: micellar electrokinetic capillary chromatography.

## 4 Conclusions

This study demonstrates the successful application of 2D-IC system for clean isocratic chromatographic separations and analyses of two NNIs in blank complex samples in basic media which proved advantageous for conversion of non-fluorescent nicotinoids namely imidacloprid and clothianidin into fluorescent specie under online post column UV irradiation in one dimension while same time other IC dimension appeared a powerful tool to discard matrix interferences of three complex food samples (honey, ginger, durian, apple, tomato and cucumber) extracts.

## Conflicts of interest

There is no conflict of interest is declared.

## Supplementary Material

RA-008-C7RA12555K-s001
