# Supplementary material for: Comprehensive two-dimensional ion chromatography (2D-IC) coupled to a post-column photochemical fluorescence detection system for determination of neonicotinoids (imidacloprid and clothianidin) in food samples
Source: RSC Adv. 2018 Mar 2;8(17):9277–86. doi: 10.1039/c7ra12555k (PMC9078649; doi:10.1039/c7ra12555k)
Supplement: RA-008-C7RA12555K-s001 [file RA-008-C7RA12555K-s001.pdf]

Electronic Supplementary Material (ESI) for RSC Advances.
This journal is © The Royal Society of Chemistry 2018

Comprehensive two-dimensional ion chromatography (2D-IC) coupled to post-column photochemical fluorescence detection system for determination of neonicotinoids in food samples

Nadeem Muhammad[a, b], Fenglian Wang[b], Qamar Subhani[b, c], Qiming Zhao[b, e], Muhammad Abdul Qadir[d], Hairong Cui[a], Yan Zhu[b,*]

[a]Department of Environmental Engineering, Wuchang University of Technology, Wuhan, 430223, China.

[b]Department of chemistry, Xixi Campus, Zhejiang University, Hangzhou 310028, China.

[c]Higher Education Department, Punjab, Lahore, Pakistan.

[d]Institute of chemistry, University of the Punjab, Lahore, Pakistan.

[e]College of Pharmacy, Zhejiang Chinese Medical University, Hangzhou 310053, China.

(*) Corresponding author:

Tel.: +86 571 88273637 Fax: +86 571 88823446.

E-mail address: zhuyan@zju.edu.cn (Y. Zhu)

Contents

1. The PIF excitation and emission spectra's of IMI and CLT in basic media and effect of difference bases on the PIF intensity of NNIs……………………………………………………………2
2. Study of effect of pH and irradiation time on PIF intensity of both NNIs ………………2

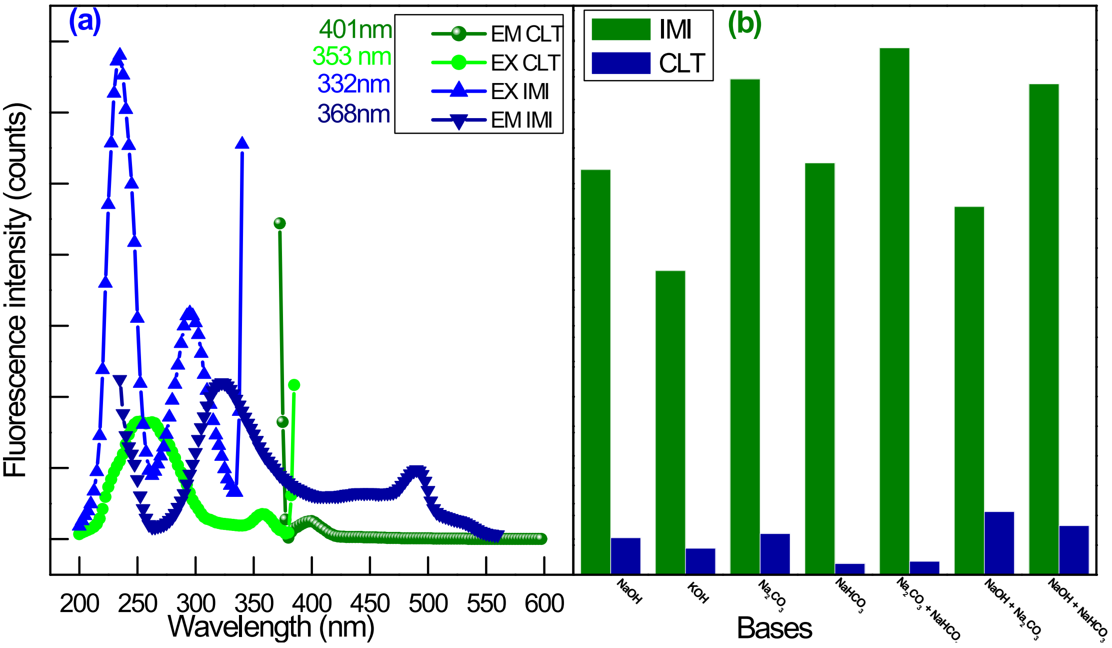

Fig. S1. (a) The PIF excitation and emission spectra's of IMI and CLT in basic mobile phase (1.5 mM Na_2CO_3 +15 mM NaOH + 18 % ACN) and (b) the PIF intensity of IMI and CLT in different bases.

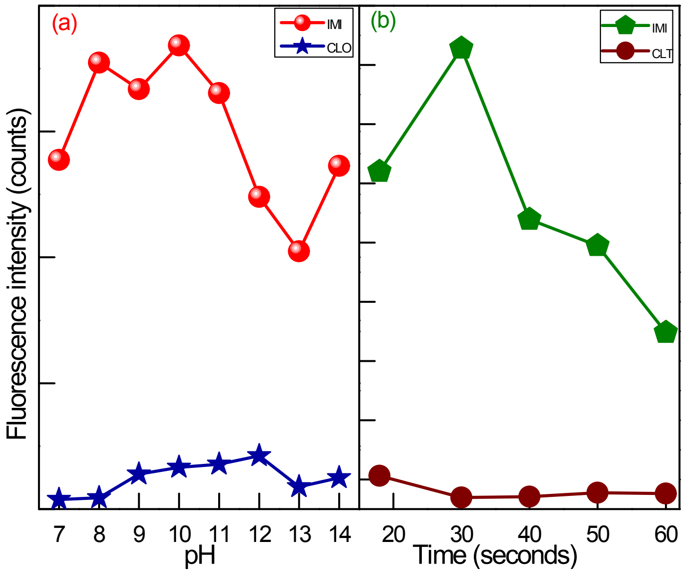

Fig. S2 (a) Effect of pH on PIF signal of IMI and CLT and (b) Effect of irradiation time on PIF intensity of IMI and CLT
